# Benefits of Dental Society Meetings

**Published:** 1896-08

**Authors:** D. E. Delzell

**Affiliations:** Logansport, Ind.


### Benefits of Dental Society Meetings.

BY DR. D. E. DELZELL, LOGANSPORT, IND.

Read before the Indiana State Dental Society, Indianapolis, Ind., June 30, 1896.

Association stimulates ambition, develops the intellect and makes one more bright and companionable. This is true of the professional as of the social side of life.

Man is a social being, he has advanced under social conditions, and there is in the professional, as well as the mental and moral life, profounder reasons for association as a means of educational and professional growth than there are for mere material ends, and the time has come when we cannot afford, even in a pecuniary sense, to ignore associations. There is a communication of mind with mind in which probably all who associate with one another participate, however unconsciously. There is not an individual, however humble, that does not have some influence, and when we put our minds in concord with others, when our hearts vibrate in harmony with them, how much we can do for the benefit of each other.

Association gives a man greater power for growth. The growing man learns something from everything he sees or hears; nothing can touch him that does not teach him. The power to grow is fed by nothing so much as keeping the mind open to every possible suggestion from every possible source—"to surrender one's self to the education of life is to receive and give, in the largest measures."

Progress is the eternal law of nature, and in this progressive age, with the rapid strides our profession is taking it is necessary for us to keep wide awake and use every energy at our command in order to keep in touch with it. It takes a better dentist to make a financial success now than it did ten years ago; and ten years hence the man who sticks to the methods now in vogue will find himself more of a back number than are the laggards of to-day.

The dental society largely contributes to the fraternal intercourse and good-fellowship of its members. The interchange of ideas through papers, discussions and clinics increases our knowledge and is a well-recognized avenue through which the standard of our profession is brought to a higher plane, to not only the members but to the world at large. Here it is that men of brains and genius professionally show their light, and what is their knowledge becomes through the dental society the knowledge of all of its members. It will be found that the leaders in any profession are the leaders in associations for the advancement of their chosen profession, be it what it may.

The ministers have their associations, the lawyers theirs; in fact, all callings in life have their societies. He who scorns associations naturally places himself beyond the reach of progress; he will get into a rut and the probabilities are that he will stay there.

Isolation dulls ambition, breeds selfishness, suspicion, distrust, until all the better feelings are blunted or destroyed.

I well remember attending the twenty-fifth anniversary of the Chicago Dental Society some years ago, and, with the exception of the meeting last year at Detroit, was one of the grandest meetings I ever had the pleasure of attending. At that meeting I saw a great many of the shining lights of the profession from almost every State. Now, to see those men and hear them talk was to me a satisfaction worth many times the cost of time and money that I sacrificed in going.

Personally, I feel that I owe much for whatever success I have attained to attendance on society meetings, Without them I know not where I would now be, and I think that if the profession generally—I mean those who are not in the habit of attending—could be brought to realize the benefit to be derived from attending these meetings, there would be many more of them in attendance than there are generally.

